# Intersectional social-economic inequalities in breast cancer screening in India: analysis of the National Family Health Survey

**DOI:** 10.1186/s12905-021-01464-5

**Published:** 2021-09-07

**Authors:** Jyotsna Negi, Devaki Nambiar

**Affiliations:** 1 Independent Consultant, 62 Stratford Rd, Kensington, California 94707 USA; 2 The George Institute for Global Health, New Delhi, India; 3 Faculty of Medicine, University of New South Wales, Sydney, Australia; 4 Prasanna School of Public Health, Manipal Academy of Higher Education, Manipal, India

**Keywords:** India, Breast cancer screening, Inequalities

## Abstract

**Background:**

Breast cancer incidence rates are increasing in developing countries including India. With 1.3 million new cases of cancer been diagnosed annually, breast cancer is the most common women’s cancer in India. India’s National Family Health Survey (NFHS-4) data 2015–2016 shows that only 9.8% of women between the ages of 15 and 49 had ever undergone breast examination (BE). Further, access to screening and treatment is unequally distributed, with inequalities by socio-economic status. It is unclear, however, if socio-economic inequalities in breast examination are similar across population subgroups.

**Methods:**

We compared BE coverage in population sub-groups categorised by place of residence, religion, caste/tribal groups, education levels, age, marital status, and employment status in their intersection with economic status in India. We analysed data for 699,686 women aged 15–49 using the NFHS-4 data set conducted during 2015–2016. Descriptive (mean, standard errors, and confidence intervals) of women undergoing BE disaggregated by dimensions of inequality (education, caste/tribal groups, religion, place of residence) and their intersections with wealth were computed with national weights using STATA 12. Chi-square tests were performed to assess the association between socio-demographic factors and breast screening. Additionally, the World Health Organisation’s Health Equity Assessment Toolkit Plus was used to compute summary measures of inequality: Slope index for inequality (SII) and Relative Concentration Indices (RCI) for each intersecting dimension.

**Results:**

BE coverage was concentrated among wealthier groups regardless of other intersecting population subgroups. Wealth-related inequalities in BE coverage were most pronounced among Christians (SII; 20.6, 95% CI: 18.5–22.7), married (SII; 14.1, 95% CI: 13.8–14.4), employed (SII: 14.6, 95%CI: 13.9, 15.3), and rural women (SII; 10.8, 95% CI: 10.5–11.1). Overall, relative summary measures (RCI) were consistent with our absolute summary measures (SII).

**Conclusions:**

Breast examination coverage in India is concentrated among wealthier populations across population groups defined by place of residence, religion, age, employment, and marital status. Apart from this national analysis, subnational analyses may also help identify strategies for programme rollout and ensure equity in women’s cancer screening.

**Supplementary Information:**

The online version contains supplementary material available at 10.1186/s12905-021-01464-5.

## Background

Cancer is the second most common cause of death globally, accounting for 9.6 million deaths in 2018, 70% of which occurred in Low- and Middle-Income Countries (LMICs) [1]. Breast Cancer (BrCa) is the leading cause of cancer mortality in women worldwide [2, 3] and burden is increasing in LMICs [4]. Although BrCa can be detected at earlier stages by simple breast examination and is treatable, most Brca cases are diagnosed very late [5]. This is particularly a matter of great concern in LMICs where BrCa often results in higher morbidity and financial constraints to households as compared to high income countries. For instance, although, the estimated number of new breast cancer cases diagnosed in the USA [6] is 1.6 times those diagnosed in India [7] in the year 2020, the estimated number of deaths due to BrCa in India is twice the deaths in the USA.^1^ With 1,78,361 new cases diagnosed and 90,408 deaths in the year 2020, BrCa is the most common form of cancer affecting women in India [7]. Nevertheless, most women remain unscreened, and late diagnosis is common: survival rates of women with BrCa range from 25.3 to 48.4% in India, much lower than other Asian countries like China (57.6–82.3%), Thailand (55.8–63.6%), and the Philippines (34.7–51.9%) [8]. India’s National Family Health Survey (NFHS-4) data 2015–2016 for the first time collected data on BrCa screening: it found that only one in ten women between the ages of 15 and 49 in India had ever undergone breast examination [9].

The goal of screening for BrCa is to identify signs of breast cancer among all women even before the symptoms appear [10]. The key to control BrCA’s outcome and improve survival rates is awareness generation and early detection to promote early diagnosis and screening of BrCa [11]. Breast cancer detected at an early stage is found to be associated with a reduction in cancer deaths across many study designs [10]. In a recent Indian trial, biannual clinical breast examinations were found to be associated with a 30% reduction in cancer mortality among those aged 50 and older [12]. In India, until 2016, there was no national population-based breast cancer screening programme [5], and most women seeking mammography went to the private sector, or had to rely on opportunistic screening under the National Programme for Prevention and Control of Cancer, Diabetes, Cardiovascular diseases and Stroke for diagnosis in the public sector [8]. In light of this, Government of India formulated a population based cancer-screening program in 2016 where all women above age 30 were eligible for regular breast, cervix and oral cancer screening [13]. Population-based screening programmes are intended to assure more equity in access in comparison with other health initiatives such as opportunistic screening programmes [3]. However, social inequalities in access can still be observed in population-based programmes [4], as disadvantaged populations are at high risk but end up being excluded [5].

Previous studies on BrCa screening published mostly in the developed world have found that several socio-economic, demographic, and geographic variables are associated with breast cancer screening. Similarly, some studies from the developing world also show that socioeconomic determinants such age, education, marital status, and income—are important determinants associated with the likelihood of receiving breast cancer screening [4, 14–20].

BrCa in its earliest stages is painless and produces masses; women from low socioeconomic groups, and/or with low levels of education tend not to seek care even when after noticing a lump for fear of facing rejection by family and community, fear of job loss, hesitancy of discussing breast cancer topic with family, fear of having to face surgery and in turn catastrophic health expenditures, fear of dying due to the disease and the notion that the condition is incurable [5]. A 2006 study in Trivandrum, Kerala found that Muslim women, unmarried women and those with professional occupation (other than manual) were less likely to undergo clinical breast examination as compared to Hindu women, married women and homemakers [16]. There is further evidence that age [21], social economic status, marital status, education [22] and health status may have an impact on the patterns of women undergoing breast screening [5, 14]. In addition, several studies among Indian women reported that religion and caste are barriers to BrCa screening uptake [23]. There is also evidence that rural women are less likely than urban women to go for breast screening [2, 24].

Most of these studies have a small sample size and do not look at the interplay of factors affecting breast cancer screening. Income has been reported as a significant determinant in undergoing breast cancer screening in almost all studies but there is no study to our knowledge that examines income inequalities within subgroups by age, education, religion, caste and other factors that may influence screening uptake. Filling this gap, in this paper we sought to examine the magnitudes and intersections of wealth related inequalities among women who reported ever undergoing breast examination in India with other dimensions of inequality.

## Methods

Our analysis sought to examine inequalities related to education, place of residence, religion, caste and tribal status, education, age, employment status and marital status in self-reported breast examination among different wealth groups using double disaggregation. Data analysed for this study was secondary data sourced from National Family Health survey (NFHS), fourth round, conducted during 2015–2016. This survey comprised a nationally representative sample of household: 699,686 women were interviewed using a multistage sampling design. NFHS 4 collected data on socio demographic characteristics of members of the households like age, education, occupation, marital status; household information such as religion, caste and tribal status, electricity, water and sanitation, insurance; as well as health related indicators like experiences with reproductive and child health service delivery, non-communicable disease related risk factors and health seeking. Information regarding ever undergone BE, an indicator for breast cancer screening coverage, was collected from women age 15–49 in each selected household. The question asked was “have you ever undergone breast examination?”. We constructed a binary variable for BE with a value of 1 if the respondent reported ever undergoing BE, and 0 if not.

### Dimensions of inequality

The dimensions of inequality selected in this paper were: place of residence, religion, caste/tribal groups, education, age, employment and marital status across wealth quintiles based on the existing literature on screening and health inequalities in India [2, 14, 16–19]. Wealth quintiles were constructed by way of a principal component analysis using an asset index of household assets such as fan, television, car, scooter, animals and land. Wealth quintiles were used as proxy to socioeconomic status of households. Four dummy variables of education attainment were created: no education, primary education, secondary education, and higher levels education. Four dummy variables of caste and tribal group were created (Scheduled Tribe, ST; Scheduled Caste, SC;’Other Backward Classes, OBC; and General) as per convention [25]. Four dummy variables of religion were created: Hindu, Muslim, Christian, and ‘Sikh, Buddhist, and others’. Age was grouped into four categories: 15–24, 25–29, 30–34, 35–49, Employment status was a binary variable: not being employed was coded as 1 and zero otherwise and last, marital status was also a binary variable where being currently married was coded as 1 and zero otherwise.

We categorised each of the seven dimensions (place of residence, religion, caste/tribal groups, education, age, employment and marital status) by wealth quintile (poorest, poor, middle, richer and richest groups) such that each group had five sub dimensions. That is, sub groups were created like religion differences among poor, following intersectional quantitative methods used in Indian datasets previously [26, 27]. This is illustratively explained for the dimension of religion in Table 1 and provided for the entire analysis in Table 2. For example, poorest Hindu women to richest Hindu women, poorest Muslim women to richest Muslim women and so on.

**Table 1:** Illustrative matrix of subgroups

Religion	Poorest (Pt)	Poor (Pr)	Middle (M)	Richer (Rr)	Richest (Rt)	Wealth difference within religion subgroups
Hindu (H)	HPt	HPr	HM	HRr	HRt	Slope Index of Inequality and Relative Concentration Indices to measure income related inequalities within respective religion
Muslim (M)	MPt	MPr	MM	MRr	MRt	
Christian(C)	CPt	CPr	CM	CRr	CRt	
Sikh/Buddhist/Others (O)	OPt	OPr	OM	ORr	Ort

**Table 2:** Demographic characteristics of women in the survey by wealth quintiles represented by N (%), NFHS 2015–2016

Dimension	Poorest	Poor	Middle	Richer	Richest
**Place of residence**					
Urban	6411 (2.6)	15,577 (6.4)	37,118 (15.3)	74,359 (30.7)	108,759 (44.9)
Rural	117,643 (25.7)	121,323 (26.5)	106,696 (23.3)	73,619 (16.1)	38,180 (8.3)
**Religion**					
Hindu	104,671 (18.6)	112,649 (20)	117,153 (20.8)	115,481 (20.5)	113,784 (20.2)
Muslim	15,551 (16.1)	18,969 (19.7)	19,648 (20.4)	23,264 (24.1)	19,028 (19.7)
Christian	1677 (10.1)	2555 (15.4)	3603 (21.7)	4158 (25)	4627 (27.8)
Other (Sikh/Buddhist etc.)	2155 (9.4)	2727 (11.9)	3410 (14.9)	5074 (22.2)	9500 (41.5)
**Caste**					
Scheduled Tribe	26,305 (41)	16,864 (26.3)	10,265 (16)	6692 (10.4)	4019 (6.3)
Scheduled Caste	32,627 (22.9)	33,918 (23.8)	32,558 (22.8)	25,910 (18.2)	17,607 (12.3)
Other Backward Caste	48,362 (15.9)	57,517 (18.9)	65,826 (21.7)	70,898 (23.3)	61,234 (20.2)
General	13,143 (8.1)	22,740 (14)	29,373 (18.1)	38,444 (23.7)	58,566 (36.1)
**Education**					
No education	70,274 (36.6)	51,568 (26.8)	37,826 (19.7)	23,412 (12.2)	9055 (4.7)
Primary	18,613 (21.3)	22,856 (26.2)	21,177 (24.3)	16,265 (18.6)	8322 (9.5)
Secondary	33,752 (10.2)	57,952 (17.5)	75,046 (22.7)	87,400 (26.4)	76,887 (23.2)
Higher	1415 (1.6)	4524 (5.1)	9766 (10.9)	20,901 (23.4)	52,676 (59)
**Age group**					
15–19 years	25,257 (20.8)	27,378 (22.5)	25,999 (21.4)	23,379 (19.2)	19,540 (16.1)
20–24 years	19,666 (16)	24,486 (19.9)	26,522 (21.6)	27,177 (22.1)	25,114 (20.4)
25–34 years	37,913 (17.9)	39,408 (18.6)	42,751 (20.2)	45,497 (21.5)	46,243 (21.8)
35–49 years	41,219 (16.9)	45,629 (18.7)	48,542 (19.9)	51,926 (21.3)	56,042 (23)
**Employment status**					
Not in workforce	11,445 (14.2)	13,951 (17.3)	15,962 (19.8)	18,545 (23)	20,846 (25.8)
Others	7271 (21.2)	7696 (22.4)	7857 (22.9)	6439 (18.8)	5057 (14.7)
**Marital status**					
Currently married	92,919 (18.2)	100,910 (19.7)	104,665 (20.5)	107,455 (21)	105,425 (20.6)
Others (unmarried-widowed-separated)	31,135 (16.5)	35,990 (19.1)	39,150 (20.8)	40,524 (21.5)	41,515 (22)

Descriptive (mean, standard errors and 95% confidence intervals) of women undergoing BE disaggregated by seven dimensions of inequality and their intersections with wealth were obtained. Chi square tests were used to find the associations between BE and selected dimensions. All descriptives were computed in STATA 12 [28] with national sampling weights for women applied using the svy command to account for varying response rates among the sampled population. In order to understand inequalities within wealth subgroups, we computed both the absolute Slope Index of Inequality (SII) and Relative Concentration Index (RCI) within each sub dimension of inequality using the World Health Organisation’s Health Equity Assessment Toolkit (HEAT) Plus [29]. SII, an absolute and complex summary measure of inequality, is regression based and calculated by regressing on health outcomes with the relative position of subgroups. On the other hand, RCI is a relative summary measure that displays the concentration of the health variable in the distribution of population ranked by wealth and was multiplied by 100 for easier interpretation. For further understanding of these summary measures, please refer to the HEAT plus technical notes [30]. We also conducted a multivariate logistic regression to identify the relationship between our dependent variable BE and selected dimensions of inequality as indicated in Additional file 1. This study did not involve human subjects research and was conducted using publicly available data.

## Results

We found that the percentage of women belonging to ‘poorest category’ in rural dwellings was 9 times greater than those in urban dwellings. The percentage of women belonging from poorest to richest quintile ranged from 3 to 45% respectively in urban areas and from 26% in poorest quintile to 8% in richest quintile in rural areas. As expected, the poorest quintile had a disproportionate concentration of uneducated, ST and unemployed women while the richest quintile had more of urban, Sikh/Buddhist/other religion, general caste, highly educated and employed women. Detailed demographic characteristics of the sample disaggregated by wealth and its intersecting with other dimensions of inequalities (place of residence, religion, caste and tribal group, education, age, employment and marital status) are presented in Table 2.

### Descriptive statistics

Mean and 95% confidence interval of those undergoing BE by wealth intersecting with other dimensions of inequalities (place of residence, religion, caste and tribal group, education, age, marital status and employment) are presented in Table 3.

**Table 3:** Descriptive analysis and summary measures for breast cancer screening coverage using NFHS 2015–2016

Dimension	Poorest	Poor	Middle	Richer	Richest	SII	RCI
**Place of residence**							
Urban	5.4 (4.4–6.4)	7.8 (6.7–9)	10.7 (9.7–11.6)	11.4 (10.8–12)	13.2 (12.6–13.8)	6.1 (5.7–6.6)	7.5 (7.3–7.8)
Rural	5.3 (5.1–5.5)	7.3 (7–7.6)	9.5 (9.2–9.8)	12 (11.6–12.5)	15.8 (15.2–16.5)	**10.8** (**10.5**–**11.1**)	**19.4** (**19**–**19.7**)
**Religion**							
Hindu	5.5 (5.2–5.7)	7.6 (7.3–7.9)	9.9 (9.5–10.2)	11.6 (11.2–12)	13.1 (12.7–13.6)	9.7 (9.4–9.9)	15.9 (15.6–16.2)
Muslim	3.9 (3.4–4.4)	5.5 (4.9–6.1)	8 (7.1–9)	10.5 (9.7–11.4)	13.6 (12.6–14.7)	12.2 (11.6–12.9)	22.2 (21.2–23.2)
Christian	5.1 (3.9–6.3)	6.2 (5.1–7.4)	10 (8.5–11.5)	14.5 (12.7–16.3)	20.6 (18.5–22.7)	**20.8** (**18.8**–**22.7**)	**24.4** (**22.8**–**25.9**)
Other	6.4 (4.7–8.2)	12.2 (9.6–14.8)	17.5 (14.8–20.2)	17.9 (15.3–20.5)	20.1 (18.5–21.7)	13.6 (11.8–15.4)	11.9 (11.2–12.6)
**Caste and tribal group**							
ST	6.4 (5.9–6.9)	9.1 (8.4–9.9)	10.2 (9.3–11.1)	11.1 (9.8–12.5)	11.8 (9.2–14.4)	7 (6.2–7.7)	12.4 (11.9–13)
SC	4.9 (4.5–5.2)	7.6 (7.1–8.2)	10.4 (9.7–11.1)	12.1 (11.2–13)	13.5 (12.4–14.7)	10.8 (10.2–11.3)	18.5 (17.9–19.1)
OBC	5 (4.7, 5.3)	6.8 (6.5, 7.1)	9.5 (9.1, 9.9)	12 (11.5, 12.5)	14.3 (13.7, 14.9)	**11.7** (**11.3**, **12.1**)	**18.7** (**18.4**, **19.1**)
General	5.3 (4.7, 5.9)	7.4 (6.6, 8.1)	9.8 (9, 10.6)	11.3 (10.6, 12)	13.8 (13.1, 14.5)	10 (9.5,10.6)	13.9 (13.5,14.3)
**Education**							
No education	5.8 (5.5, 6.1)	8.4 (8, 8.8)	10.8 (10.2, 11.4)	12.4 (11.6, 13.2)	14 (12.7, 15.3)	9.5 (9, 9.9)	16.9 (16.5, 17.3)
Primary	5.3 (4.9, 5.7)	8.5 (7.9, 9.1)	11.6 (10.8, 12.5)	13.4 (12.4, 14.3)	15.9 (14.4, 17.3)	12.1 (11.3, 12.8)	**18.6** (**18**, **19.2**)
Secondary	4.3 (4, 4.6)	6.2 (5.9, 6.6)	9.2 (8.8, 9.6)	11.8 (11.3–12.3)	14.2 (13.6–14.8)	11.9 (11.6–12.3)	18.5 (18.1–18.9)
Higher	3.6 (2.4–4.8)	4.3 (3.5–5.1)	6.5 (5.8–7.3)	9.3 (8.6–9.9)	13.1 (12.4–13.7)	**12.4** (**11.5**–**13.3**)	13.5 (13–14)
**Age group**							
15–19	1.5 (1.3–1.7)	2.2 (1.9–2.4)	2.4 (2.1–2.7)	2.4 (2.1–2.7)	3.1 (2.5–3.6)	1.6 (1.3–2)	11.5 (10.8–12.2)
20–24	5 (4.6–5.4)	6.6 (6.1–7.1)	8.1 (7.4–8.7)	8.5 (7.9–9.1)	7.8 (7.2–8.3)	3.4 (2.9–3.9)	7.4 (7.2–7.7)
25–34	6.5 (6.1–6.9)	9.2 (8.8–9.7)	12 (11.4–12.5)	13.9 (13.2–14.5)	15.6 (14.9–16.3)	11.4 (10.9–11.9)	15.4 (15.1–15.7)
35–49	6.6 (6.3–6.9)	9.3 (8.9–9.7)	12.8 (12.2–13.3)	15.7 (15.1–16.3)	19 (18.3–19.7)	**15.8** (**15.3**–**16.3**)	**18.9** (**18.5**–**19.3**)
**Employment status**							
Not in workforce	5.2 (4.6–5.8)	6.5 (5.8–7.1)	10.1 (9.3–10.9)	11.6 (10.8–12.4)	14.4 (13.4–15.4)	11.8 (11.1–12.6)	**18** (**17.4**–**18.7**)
Others	7 (6.3–7.8)	9.7 (8.6–10.7)	12.7 (11.3–14.1)	13 (11.6–14.4)	16.3 (14.4–18.1)	**14.6** (**13.9**–**15.3**)	12.4 (11.5–13.3)
**Marital status**							
Currently married	6.3 (6.1–6.6)	9.1 (8.8–9.4)	12.1 (11.6–12.5)	14.7 (14.2–15.2)	17.6 (17–18.2)	**14.1** (**13.8**–**14.4**)	**18.3** (**18–18.6**)
Other	2.1 (1.9–2.3)	2.5 (2.3–2.7)	3.7 (3.4–4)	3.7 (3.4–4.1)	4.5 (4.1–4.9)	3.0 (2.7–3.3)	13.7 (13.2–14.3)

All subgroups were statistically significant at *p* < 0.05 and values have been rounded to one decimal place. The largest magnitude of wealth-related inequality for each dimension has been bold

**Fig. 1:**
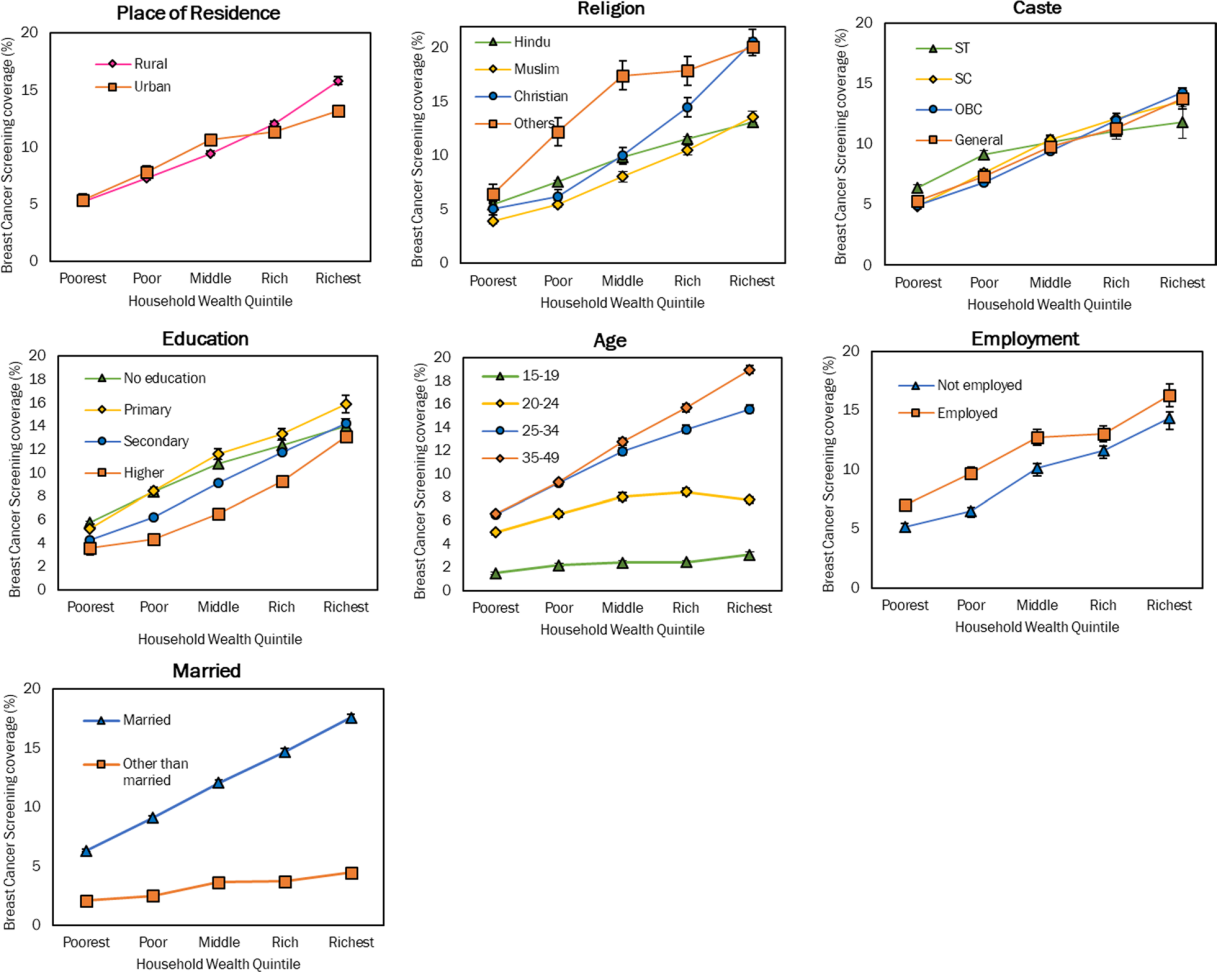
BE coverage (%) disaggregated by dimensions of inequality

Overall, about 9.7% of women aged 15–49 had ever undergone a breast examination. This percentage varied by different socio-economic dimensions. Poorest to Richest (q_1_–q_5_) gap in BE coverage in rural India was 10.6 percentage points while it was 7.8 percentage points in urban India (see Fig. 1 and Table 3). BE coverage was 3 times higher in rural richest quintile than the rural poorest quintile while this ratio was 2.5 in urban areas. BE coverage was lowest among Muslims and highest among ‘other’ religion in the poorest quintile. The Absolute q_5_-q_1_ difference was highest among Christians (15.5), followed by married women (11.3) and lowest among 15–19 age group (1.6), ‘other than married’ (2.4). The absolute q_5_–q_1_ difference in BE coverage among ‘OBC group’ was 9.3 percentage points (highest amongst all caste groups) and the ratio was 2.9 indicating three times higher coverage in the richest quintile than the poorest quintile. The pattern by education, in contrast, was mixed: the poorest women with higher education had lower BE coverage (3.6%, 95% CI: 2.4, 4.8) and the wealthiest women with primary education had highest BE coverage (15.9%, 95% CI: 14.4, 17.3). The absolute q_5_–q_1_ difference (10.6) was highest among women with primary education while the ratio (3.6) was highest in women with higher education levels. The Absolute q_5_–q_1_ difference (12.4) in age group for BE coverage was highest in the 35–49 age group and the ratio was 3 indicating that the richest quintile had three times higher BE coverage than the poorest quintile. As expected, BE coverage was lowest among those aged 15–19 across all quintiles. Employed women reported greater BE coverage than unemployed women across bottom three quintiles. The absolute q_5_–q_1_ difference in BE coverage by employment was insignificant in top two quintiles. BE Coverage among currently married women ranged from 6.3% in poorest quintile to 17.6% in richest quintile as compared to 2.1% in poorest quintile to only 4.5% in richest quintile among those not married. Among married women, BE coverage was three times higher in richest quintile than poorest quintile while among ‘other than married’ quintile, it was two times higher.

## Summary measures of inequality

When looking at summary measures, we found statistically significant  wealth inequality in breast examination across all intersecting subgroup dimensions. All the SIIs and RCIs were positive, meaning that breast examination coverage was concentrated among wealthier quintiles regardless of place of residence, religion, caste and tribal group, education, age, employment status and marital status (Table 3).

Our absolute and relative measure were generally consistent with each other (see Table 1). Looking at SlI, the greatest wealth-related inequalities in breast examination coverage were seen among Christians (SII: 20.6, 95% CI: 18.5–22.7). Across other dimensions, we saw significantly greater inequality in rural areas (SII: 10.8, 95% CI: 10.5–11.1), among SC (SII: 10.8, 95% CI: 10.2–11.3), OBC (SII: 11.7, 95% CI: 11.3–12.1), and other general populations (SII: 13.9, 95% CI:13.5–14.3), as compared to ST groups (SII: 7.0, 95% CI: 6.2, 7.7), among groups with education (SII: 12.4, 95% CI:11.5, 13.3), as compared to those without (SII: 9.5, 95% CI: 9.0, 9.9), among those aged 25–34 (SII: 11.4, 95% CI: 10.9, 11.9), and 35–49 (SII: 15.8, 95% CI: 15.3, 16.3), as compared to younger populations, among the employed (SII:14.6, 95% CI:13.9, 15.3), compared to those not, and those married as compared to those not (SII: 3.0, 95% CI:2.7, 3.3). We saw the same general pattern with the RCI for place of residence, religion, caste and tribal group, age-group and marital status, although for education, the magnitude of wealth-related inequality in BE coverage among primary & secondary educated women was higher relative to those with higher levels or no of education. We also saw a higher magnitude of wealth related inequality in BE coverage among those not in the workforce in comparison to those who were.

## Discussion

To best of our knowledge, this study is the first of its kind which measures inequality in BE coverage using double disaggregation. We examined wealth-related inequalities in BrCa screening coverage of Indian woman intersecting with place of residence, religion, age, employment and marital status. One of the most interesting findings of our study was that BE coverage was concentrated among wealthier groups across all population subgroups. This strongly relates with the fact that a woman’s economic status largely influences her decision and ability to access screening.

Importantly, the magnitude of wealth-related inequality varied by sub-dimensions. Some studies conducted in upper and middle income countries have reported that marital, socio- economic level and educational status have significant associations with the utilization of BrCa screening services [31–34]. We found large wealth related inequalities among Christians, Muslims, rural women closely followed by 35–49 age group, OBC and SC groups, primary and secondary educated groups, married and unemployed groups.

Higher Income levels has been reported as an important factor for uptake of BrCa screening services in most of the studies but their interplay with different social subgroups is complex. The results of this study show that although residing in rural areas is associated with greater BE coverage when compared to residing in urban areas, yet higher wealth related inequality persists among rural women in comparison to urban women. A study assessing social determinants in BrCa screening among women of age 40–69 years from 15 developing countries found that among women residing in rural areas, middle socio economic status (SES) household had reduced likelihood of BrCa screening in comparison to high SES household [35]. A study using the same data source as ours, assessing BrCa screening uptake in districts found that residing in rural areas in addition to being married, belonging to general caste and higher income status contribute positively to utilization of BrCa screening services [2]. A cluster randomised controlled cohort study in Mumbai reported that increasing age, Muslim religion, higher education, higher-income, single unmarried women were identified as predictors for non-compliance to screening [18].

We also found increasing age was significantly associated with the uptake of undergoing breast examination in our study but a coverage reported among the younger age group in our study [15–25] may be either suggestive of margin of error in self-report of BE or instrumentation issues, as the likelihood of a BE in this age group is extremely low. Additionally, in the present study, we found that Muslim and Christian women had highest wealth-related inequality in BE coverage, with coverage concentrated among wealthier populations. Cultural and religious beliefs often interweave to form distinctive traditions and rules which affect women’s decision to participate in screening [36, 37]. These may be more concentrated among poorer households as compared to wealthier households, resulting in a wealth gradient. In a breast cancer screening trial conducted in 2006 in Trivandrum, Kerala, India, findings were similar to our study: adjusted results showed that Christians were about 40% less likely to attend breast clinics than Hindus [16]. This study also reported that women who were not currently married were significantly less likely to participate in any level of screening process than married women. Another cross sectional study in a district in Kerala interviewing 809 women found that age 35–50 years, marriage, and employment were significant predictors of uptake in BrCa screening [19]. Findings from a systematic review of BrCa screening uptake in LMICs showed that religion, education, lack of accessibility, lack of knowledge about the diseases and screening were considerable barriers to BrCa screening in women [15].

In most of the studies, being married and employed were found to be associated with an increase in utilization of BrCa screening. corroborating the results of our study. However, it is also important to note that we also found large magnitudes of wealth-related inequalities in BE coverage among those married or employed suggesting that screening is concentrated among the well-off women from these subgroups. This is of concern because a recent systematic review found that non-married women are at greater risk of BrCa [38].

Women with primary and secondary education levels had higher magnitude of relative wealth-related inequality in BE coverage compared to those with higher education while the absolute inequalities  were nearly the same. The literature suggests that households with less education may have lower awareness of the advantages of getting screened for cancer [35]. Importantly, the study showed that magnitude of inequalities may differ when measuring them in absolute or relative terms. Additionally, a qualitative study conducted in rural Andhra Pradesh to understand physician’s perspective on screening methods followed by women diagnosed with breast cancer reflected that awareness of screening is limited to higher socioeconomic groups [20]. A community-based study where screening programme of women age 30–64 was implemented in urban slums of Mumbai, India found that literacy was a positive predictor of participation in screening while belonging to Muslim religion was a negative predictors of participation in screening [17, 18]. The interplay of education and wealth status is under-explored in the literature and warrants further study in relation to BrCa.

A previous study has also reported economic status and education as leading predictors of participation in BrCa screening [35], although their interplay was not explored. A systematic review conducted in 2017 examining BrCa screening barriers reported lack of breast cancer knowledge, and an inadequate understanding of the screening role as the key barriers of women’s screening participation in LMICs, noting also that BrCa is not a health priority in LMICs, resulting in screening programmes being either opportunistic or not present at all [15]. As aforementioned, population-based screening for BrCa in India was put in place only after 2016. The fifth round of NFHS is in the process of being completed; it will be important to see how the magnitude of socio-economic inequalities has changed after the launch of the national screening program. Routine screening may be a significant intervention to control BrCa and improve outcomes, especially in LMICs like India where BrCa mortality is high, and access to mammography is much lower compared to high income country settings. As screening access is sought to be improved, it will be important to ensure socio-demographic, socio-economic, geographic and other inequalities do not fragment this access.

There are some limitations in this study. Firstly, the age group of study is restricted to 15–49-year-old women because NFHS provides data for this reproductive age group only (In the 15–19 age group, screening was reported—3% of the youngest age group [15–24], suggestive of non-sampling and instrumentation errors). Secondly, the data did not enable us to differentiate between who got screened for preventive purposes and who got screened after getting the disease itself. Thirdly, the data did not allow us to differentiate between women receiving screening through the government initiatives and those getting screening willingly. Further, in this analysis, we examined inequalities, but without data on consent and choice, we were not able to ascertain whether inequalities were unjust or preventable, and thus unable to take a view on whether they represent inequities.

## Conclusions

Breast examination coverage in India is concentrated among wealthier populations across populations groups defined by place of residence, religion, age, employment and marital status. Apart from this national analysis, subnational analyses may also help with identifying strategies for programme rollout and to ensure equity in women’s cancer screening. Findings further suggest that more culturally appropriate awareness campaigns especially among low socioeconomic households are needed to increase screening participation and compliance. There is also a need to monitor gaps in coverage as the ongoing population-based screening programme advances.

## Supplementary Information

**Additional file 1**. Results of multiple logistic regression analysis of Breast Cancer Screening

## Data Availability

All datasets used for supporting the conclusions of this paper are free to download using a short registration form at demographic health surveys website: at https://www.dhsprogram.com/data/Using-DataSets-for-Analysis.cfm#CP_JUMP_14037.

## Abbreviations

NFHS: National Family Health Survey
BE: Breast examination
SII: Slope index for inequality
RCI: Relative Concentration Index
BrCa: Breast cancer
ST: Schedule tribe
SC: Scheduled caste
OBC: Other backward classes
HEAT: Health equity assessment toolkit

## Footnotes

1 In 2020, an estimated 276,480 and 178,361new BrCa cases were diagnosed in the USA and India respectively. The estimated deaths due to BrCA were 42,170 in the USA and 90,408 in India.
