# Supplementary material for: Intersectional social-economic inequalities in breast cancer screening in India: analysis of the National Family Health Survey
Source: BMC Womens Health. 2021 Sep 7;21:324. doi: 10.1186/s12905-021-01464-5 (PMC8424809; doi:10.1186/s12905-021-01464-5)
Supplement: Supplementary file 1 — Additional file 1. Results of multiple logistic regression analysis of Breast Cancer Screening [file 12905_2021_1464_MOESM1_ESM.pdf]

Additional file1

Results of multiple logistic regression analysis of Breast Cancer Screening

Multivariate logistic regression analysis displayed highest odd ratios for marital status: married women were 2.46 times more likely to undergo BE compared to not married (single, separated, divorced) women. Lower the level of wealth, the less likely women were to undergo BE (compared to richest quintile: richer women were 20% less likely and poorest women were 60% less likely to undergo BE). Women who had secondary education, employed, belong to schedule caste and Hindu religion had greater odds of undergoing BE by 31%, 12%, 15% and 6% respectively (see Table A1).

Table A1: Results of multiple logistic regression

Dimension	OR (95% CI)	p value
**Place of residence (Ref: urban)**		
Rural	1.06 (0.98,1.14)	0.16
**Religion (Ref: Muslim)**		
Hindu	1.06 (0.96,1.17)	0.27
Christian**	1.43 (1.2,1.71)	0
Sikh, buddhisht, others**	1.62 (1.39,1.9)	0
**Caste and Tribal Group (Ref: General)**		
SC**	1.15 (1.02,1.29)	0.02
ST**	1.07 (0.97,1.18)	0.2
OBC*	1.08 (1,1.18)	0.06
**Education (Ref: no education)**		
Primary**	1.21 (1.09,1.33)	0
Secondary**	1.31 (1.2,1.42)	0
Higher**	1.25 (1.09,1.42)	0
**Age grouo (Ref: 35-45)**		
15-19**	0.24 (0.2,0.29)	0
20-24**	0.59 (0.53,0.66)	0
25-34**	0.85 (0.79,0.91)	0
**Employment (Ref: not in workforce)**		
Employed**	1.12 (1.05,1.19)	0
**Wealth (Ref: Richest)**		
poorest**	0.39 (0.35,0.45)	0
poor**	0.51 (0.46,0.57)	0

middle**	0.75 (0.68,0.83)	0
Richer**	0.81 (0.74,0.88)	0
**Marital Status (ref: currently not married)**		0
Currently Married**	2.46 (2.18,2.77)	

Legends: OR Odds Ratio, 95%CI 95% Confidence Interval
**p<.05, variable category with p<0.05
*p<.10, variable category with p<0.10
